# Biomechanical Characterization of Unilateral and Bilateral Posterior Lumbar Interbody Fusion Constructs

**DOI:** 10.1155/2022/7081238

**Published:** 2022-08-13

**Authors:** Xiangping Peng, Shaoqing Li, Sidong Yang, Isaac Swink, Jake Carbone, Boyle Cheng, Zhanyong Wu

**Affiliations:** ^1^Department of Orthopedic Surgery, Xingtai General Hospital of North China Medical Health Group, Xingtai 054000, China; ^2^Department of Spine Surgery, The Third Hospital of Hebei Medical University, Shijiazhuang 050051, China; ^3^Department of Neurosurgery, Allegheny-Singer Research Institute, Pittsburgh, USA

## Abstract

**Objectives:**

To compare the biomechanical stability of two-level PLIF constructs with unilateral and bilateral pedicle screw fixations.

**Methods:**

Six cadaveric lumbar segments were evaluated to assess biomechanical stability in response to pure moment loads applied in flexion-extension (FE), lateral bending (LB), and axial rotation (AR). Each specimen was tested in six sequential configurations: (1) intact baseline; (2) facetectomy; (3) unilateral pedicle screws (UPS); (4) bilateral pedicle screws (BPS); (5) unilateral pedicle screws and cage (UPSC); and (6) bilateral pedicle screws and cage (BPSC).

**Results:**

Significant reductions in motion were observed when comparing all instrumented conditions to the intact and facetectomy stages of testing. No significant differences in motion between UPS, BPS, UPSC, or BPSC were observed in response to FE range of motion (ROM) or neutral zone (NZ). ROM was significantly higher in the UPS stage compared to BPS in response to LB and AT loading. ROM was significantly higher in UPSC compared to BPSC in response to LB loading only. Similarly, NZ was significantly higher in UPSC compared to BPSC in response to only LB loading. In response to AT loading, ROM was significantly higher during UPS than BPS or BPSC; however, no significant differences were noted between UPSC and BPSC with respect to AT ROM or NZ.

**Conclusion:**

BPS fixation is biomechanically superior to UPS fixation in multilevel PLIF constructs. This was most pronounced during both LB loading. Interbody support did contribute significantly to immediate stability.

## 1. Introduction

Thoroughly understanding the biomechanical characteristics of the lumbar spine is critical to furthering the treatment of spinal pathologies. One such treatment includes the use of pedicle screws and rods for spinal stabilization. This approach is widely popular for single and multilevel spinal fusions for various lumbar disorders. Several posterior fixation techniques are currently available to promote spinal fusion with bilateral fixation being considered the “gold standard” [1]. This is a result of its ability to improve arthrodesis rates, increase fusion, and prevent nonunion. Posterior lumbar interbody fusion (PLIF) also has the ability to decompress the dural sac and nerve roots while maintaining disc height and increases the rate of recovery from spinal fusion procedures [2].

While considered the gold standard in treatment, there are some drawbacks associated with the use of rigid fixation during spinal procedures. Lumbar spinal fusion has been shown to increase the rate of degeneration of lumbar segments adjacent to the instrumentation and has the potential for issues involving the device (i.e., subsidence and migration) and osteoporosis [3]. This may be due to changes in the distribution of forces with the use of stiff bilateral constructs, which have the potential to offload the disc space and thus reduce bony formation according to Wolf's Law. Numerous techniques have been studied in order to combat these deleterious effects. For example, minimally invasive approaches—such as the use of unilateral pedicle screw fixation—have the potential to increase graft loading while decreasing operating time, intraoperative blood loss, recovery time, and the risk of adjacent segment disease [4, 5]. One of the major advantages of unilateral fixation is the decrease in patient morbidity. Due to less instrumentation, there is a decrease in OR time, blood loss, and hospital stay associated with unilateral constructs [6]. The two major differences between the surgical approaches for unilateral and bilateral pedicle screws are in the amount of instrumentation used and the size of the operative field. Bilateral fixation requires dissection of both paravertebral muscles and insertion points. This is opposed to the single dissection required for unilateral fixation which allows for less tissue damage and potentially earlier functional recovery [7]. However, there is limited data comparing UPS to standard of care procedures. In addition, the data available is contradictory insofar that some studies state that unilateral fixation is as effective as bilateral fixation when used with interbody devices, while other studies have demonstrated better fixation results associated with bilateral fixation [8–11]. While it is unclear whether unilateral and bilateral fixations with interbody fusion are equitable and both sufficient for spinal fusion, limited studies have been able to accurately compare the procedures using pure moments.

Our objective was to determine if unilateral pedicle screw fixation can provide stability comparable to that of bilateral pedicle screw fixation. More specifically, the goal of this study was to investigate the stability of multilevel PLIF constructs with both UPS and BPS in response to pure moment loading.

## 2. Methods

**Figure 1:**
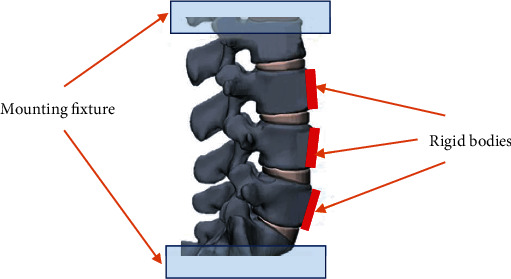
Illustration of specimen preparation showing placement of mounting fixtures at L2 and S1 as well as rigid tracking bodies attached to the anterior aspect of L3, L4, and L5.

**Figure 2:**
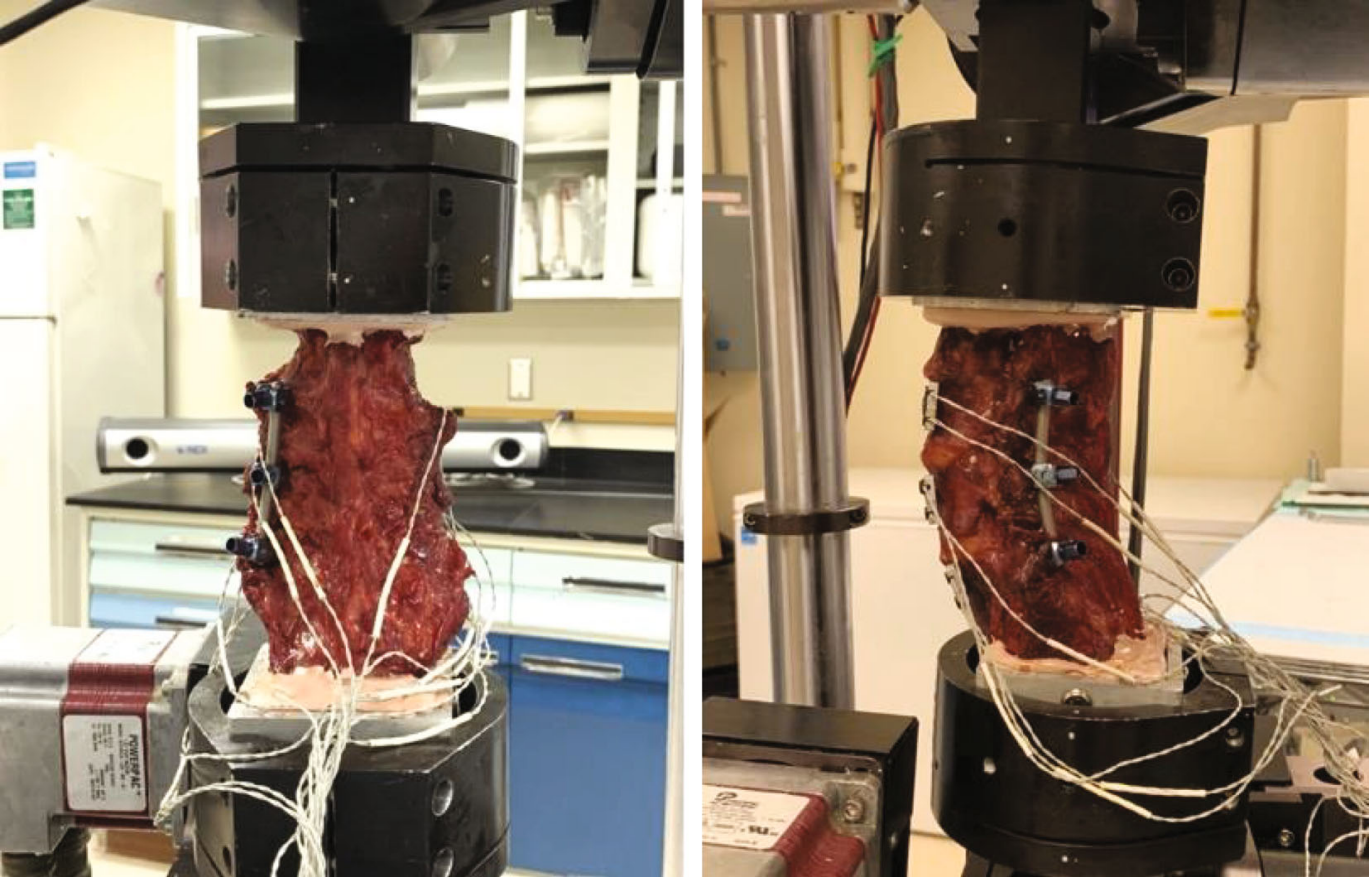
Illustration of specimen with L3-L5 unilateral pedicle screw fixation which was tested in Bose six-degrees-of-freedom spine testing apparatus.

Six cadaveric lumbar segments were obtained. Specimens were then cleaned and dissected down to osteoligamentous structures and disarticulated at the L1-L2 index level to produce L2-S1 vertebral segments for testing. Once thoroughly cleaned, an intact vertebral column was potted into custom aluminum potting rings using a thermosetting polymer, polyester resin, and hardener (Bondo, 3M, Atlanta, GA) as seen in Figure 1. After the polymer was allowed to cure, each segment was loaded into a Bose six-degrees-of-freedom spine testing apparatus and subjected to a series of pure moment flexibility tests in flexion-extension (FE), lateral bending (LB), and axial torsion (AT) modes of loadings as described by Cook et al. [12].  Figure 2 shows how this works.

The initial testing condition is referred to as Intact and is used to characterize the functional spinal unit's (FSU) baseline biomechanical characteristics before surgical intervention and instrumentation. Treatments were randomized to either the left or the right side of the segment according to Table 1. The second stage of testing, hereby referred to as Facetectomy for the remainder of this paper, involved a unilateral facetectomy followed by flexibility testing and was intended to represent the destabilized condition. The L3-L5 index levels were then instrumented in a series of procedures in both the unilateral and bilateral states. Flexibility tests were performed for each level in order to determine the efficacy of the pedicle screw constructs with and without intervertebral cages. The constructs were then tested in order from least destructive to most destructive states. Unilateral pedicle screw instrumentation (UPS) was completed immediately following the completion of unilateral Facetectomy testing, followed by bilateral pedicle screws (BPS), unilateral pedicle screws with a PLIF cage (UPSC), and finally bilateral pedicles screws with a cage (BPSC). The 6 specimens were sequentially measured on intact, unilateral facetectomy, UPS, BPS, UPSC, and BPSC. The sets of data of flexion-extension, lateral bending, and axial rotation were measured at each stage. All tested constructs were imaged via an O-arm (Medtronic) to confirm pedicle screw and cage placement.

The posterior vertebral fixation construct utilized over the course of this study was the CD Horizon Legacy Spinal System (Medtronic, Minneapolis, MN). Each construct was composed of six pedicle screws, ranging from size 5.5 × 40 mm to 6.5 × 50 mm as designated in Table 1. 5.5 × 70 mm CD Horizon Legacy titanium rods were implemented into each construct with the exception of one larger specimen, for which a 55 × 100 mm Zodiac titanium rod (Alphatec, Carlsbad, Ca) was used. All of the interbody devices used came from the Capstone Spinal System (Medtronic, Minneapolis, MN). The intervertebral cage size information for each specimen can also be found in Table 1. All screws, rods, and interbody devices were placed by a trained spinal surgeon according to the manufacturer's protocol recommendations.

All biomechanical testing was conducted using a six-degrees-of-freedom spine tester (Bose, Smart Test Series, Eden Prairie, MN) under a standard flexibility protocol with independent motors driven in load control. This characterizes the FSU's baseline flexibility and allows each specimen to serve as its own control. The flexion-extension and lateral bending protocols apply a uniform pure moment across the specimen through counteracting superior and inferior mounted stepper motors [13]. The magnitude for the pure moment protocol has been referenced in numerous peer-reviewed articles dealing with in vitro cadaveric studies [13, 14] as well as finite element analysis [15]. Each specimen was subjected to 7.5 Nm pure moment loads in flexion-extension, lateral bending, and axial torsional with no compressive preload. All samples were not damaged by the pure moment load of 7.5 Nm during the test.

During flexibility testing, the loads for FE, LB, and AT were applied for three cycles with the last cycle being use for data analysis. The range of motion (ROM) of each segment was measured using an optoelectric tracking system (Opotrak Certues, Northern Digital, Waterloo, ON) with rigid bodies fixed to the anterior aspect of the L3, L4, and L5 vertebral bodies. The rigid component, known as a Tracking Body, houses four light emitting diodes (LEDs) and was attached to the anterior surface of each vertebral body prior to each test. Four points defining the orientation of the test apparatus motor axes were digitized relative to the L3 and L5 Tracking Bodies to form an anatomically relevant coordinate system. Positional data from each rigid body were used to calculate the relative angular motion (ROM) and neutral zones (NZ) between the L3-L4 and L4-L5 disc spaces.

For statistical analysis, the ROM and NZ for each level at each stage were normalized to the intact condition. Changes in ROM or NZ from stage to stage are therefore presented as percent changes relative to the intact condition. A repeated measure ANOVA with Bonferroni post hoc analysis was performed to elucidate statistically significant differences between cohorts.

## 3. Results

**Figure 3:**
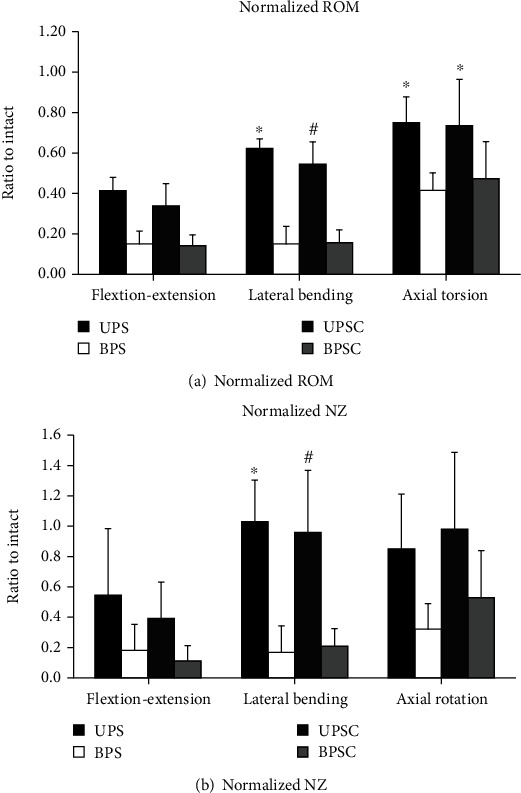
Results of flexibility testing normalized to the intact condition: (a) range of motion results for each stage of testing; (b) neutral zone results for each stage of testing. ^∗^Denotes significant difference compared to BPS; ^#^denotes significant difference compared to BPSC.

All present data can be seen in Figure 3. The data shows significant reduction in motion for all instrumented cadaveric spines with respect to the intact and facetectomy stages of testing, indicating solid fixation for all treatments. O-arm images were reviewed for confirmation of proper pedicle screw and cage placement. There were no signs of breach in any of the instrumented spines.

Descriptive statistics for flexion-extension loading can be seen in Table 2. Sphericity was violated, *χ*^2^ (14) = 51.354, and degrees of freedom were therefore corrected using the Greenhouse-Giesser method (*ε* = 0.314). This revealed a significant difference between the cohorts with respect to normalized FE ROM, *F*(1.57, 17.29) = 36.709, *p* = 0.000. Post hoc analysis showed significant decreases in motion from the Facetectomy stage of testing to all instrumented conditions; however, there were no significant differences in motion between unilateral and bilateral constructs with or without the implanted cage. Mauchly's test of sphericity indicated that sphericity was violated for the FE neutral zone data as well as *χ*^2^ (14) = 53.03, and degrees of freedom were corrected using the Greenhouse-Giesser method (*ε* = 0.296). The repeated measure ANOVA according to the Greenhouse-Giesser correction showed no significant difference between the cohorts with respect to normalized FE NZ, *F*(1.48, 16.304) = 2.37, *p* = 0.135.

Descriptive statistics for lateral bending loading can be seen in Table 3. Mauchly's test of sphericity indicated that sphericity was violated for both ROM and NZ, *χ*^2^ (14) = 47.1, *p* = 0.000 (*ε* = 0.45) and *χ*^2^ (14) = 45.43, *p* = 0.000 (*ε* = 0.467), respectively. The repeated measure ANOVA revealed a significant difference between the cohorts with respect to normalized LB ROM, *F*(2.338, 25.717) = 17.41, *p* = 0.000. Angular motion during the UPS stage of testing was significantly larger than during the bilateral stage of testing, *p* = 0.000, or the BPSC stage, *p* = 0.000. Similarly, ROM during the UPSC stage was significantly higher than the BPS, *p* = 0.000, or BPSC, *p* = 0.000, stages of testing. The repeated measure ANOVA revealed a significant difference between the cohorts with respect to normalized LB NZ, *F*(1.761, 14.087) = 45.394, *p* = 0.000. Review of post hoc analysis showed a significantly higher NZ during the UPS stage compared to the BPS stage (*p* = 0.000) or BPSC (*p* = 0.000). UPSC also demonstrated significantly larger NZ than both BPS (*p* = 0.0004) and BPSC (*p* = 0.0001).

Descriptive statistics for Axial Torsion loading can be seen in Table 4. Using the Greenhouse-Gieser correction due to violation of sphericity, the repeated measure ANOVA revealed a significant difference between the cohorts with respect to normalized AT ROM, *F*(1.843, 20.283) = 23.079, *p* = 0.000. Angular motion during the UPS stage of testing was significantly larger than during the bilateral stage of testing (*p* = 0.000) or the BPSC stage (*p* = 0.036). Similarly, ROM during the UPSC stage was significantly higher than the ROM measured during the BPS stage (*p* = 0.005); however there was no significant difference between the UPSC and BPSC stages. The repeated measure ANOVA revealed a significant difference between the cohorts with respect to normalized AT NZ, *F*(1.638, 18.02) = 10.298, *p* = 0.0017. Nonetheless, these significant differences were between the Facetectomy stage and all instrumented conditions, and there were no significant differences between instrumented cohorts. In addition, the BPS stage of testing showed significantly lower NZ than the intact condition. Figure 3 below illustrates these results.

## 4. Discussion

Posterior fusion procedures of the lumbar spine attempt to stabilize vertebral segments in order to create optimal conditions for arthrodesis to occur. These procedures are often performed with the utilization of rods and screws to immobilize the intervertebral space and allow fusion. In some instances, a surgeon may place an interbody cage within the intervertebral disc space to further promote immobilization of the spinal segment and encourage bone growth. There are a wide variety of approaches that can be utilized in order to place the interbody cage, and each has their own set of advantages and disadvantages [16]. One of the most common procedures performed during spinal fusion surgeries is the PLIF. During a PLIF, a surgeon must introduce unilateral or bilateral facet injury in order to allow the placement of the interbody device. Because of the destructive nature of this procedure, it is generally recommended that surgeons combine the use of a posterior interbody cage with pedicle screw fixation [17]. As this is the case, we aimed to explore whether or not unilateral pedicle screw fixation was equivalent to bilateral fixation when performing multilevel posterior lumbar fusion procedures.

The results of this study showed variations in construct stability based on the direction of applied load. The most pronounced differences between the UPSC and BPSC cohorts were observed during LB loading, with significantly higher ROM (59% vs. 24% of intact) and NZ (98% vs. 36% of intact) in the UPSC group compared to the BPSC cohort. Unsurprisingly, ROM and NZ measured in response to lateral bending loading were significantly higher when comparing unilateral and bilateral pedicle screw fixations without the inclusion of an interbody cage. Significant differences were also observed in axial loading conditions, with significantly higher angular motion measured during UPS testing compared to BPS constructs; however, differences between the UPSC and BPSC cohorts were insignificant. There were no significant differences observed with respect to both ROM and NZ measured during FE loading. Furthermore, the addition of a unilateral standard PLIF cage did not have a significant effect on stability in any of the tested conditions.

While in vitro cadaveric studies comparing the stability of unilateral and bilateral PLIF constructs are limited, these results correlate well with those reported in the literature. A 2008 study conducted by Yucesoy et al. showed significant reductions in the stability of two-level unilateral constructs in comparison to bilateral constructs, with lateral bending as the clear weakness of unilateral constructs [11]. Finite element models aimed at comparing the stability of unilateral and bilateral PLIF constructs have also yielded similar conclusions [6, 8]. Perhaps the most valid comparison to the present work is the finite element model presented by Kim et al. comparing the stability of unilateral and bilateral constructs with a hemilaminectomy. In this study, the authors report the ranges of motion for unilateral constructs in response to FE, LB, and AT loads of 32%, 31.7%, and 61.7% of the intact motion compared to reductions to 8%, 26.8%, and 50% of the intact motion for bilateral constructs. Another finite element study conducted by Ambati et al. produced similar results; showing reductions in angular motion to 50% of intact left bending motion and 63% of axial rotation for unilateral constructs compared to values reported for bilateral constructs of 10% of intact motion for left bending and 10% of motion for axial rotation. Furthermore, Ambati et al. found that the shape and position of the interbody device did not have a significant influence on the reported ROM [8]. This is similar to our results, which show that the addition of an interbody cage did not significantly influence the ROM or NZ at the index level. These results indicate that the stability of PLIF constructs is primarily driven by posterior instrumentation. In contrast, studies focused on comparing unilateral and bilateral fixation in conjunction with larger interbody devices such as lateral cages have shown comparable stability regardless of posterior fixation [10].

Numerous clinical studies have been published in the last decade comparing the efficacy of unilateral and bilateral PLIF and TLIF constructs, underscoring the importance of a sound biomechanical understanding of each treatment modality [5, 9, 18–21]. Results of these studies are not as clear as those biomechanics studies previously discussed in terms of establishing the superiority of unilateral or bilateral pedicle screw fixation. Many studies report similar fusion rates, complication rates, and patient reported outcomes with improvements in perioperative measures such as blood loss, OR time, and length of stay [18, 19, 21]. Those studies which included cost analysis of the two fixation strategies demonstrated significant reductions in medical expenses, with an average expense of $3,500 USD for unilateral pedicle screw fixation compared to $4,800 for bilateral pedicle screw fixation [21]. However, a 2012 study conducted by Duncan et al. contradicts these results, concluding that unilateral TLIF constructs have a higher propensity for cage migration compared to bilateral constructs (23% vs. 11%, respectively) [9]. Finally, a series of in vivo dynamic motion measurements conducted on 13 patients with unilateral pedicle screw fixation and 15 patients with bilateral pedicle screw fixation conducted by Nie et al. provides a bridge between clinical and biomechanics studies. This study showed that the increase in axial rotation observed in biomechanics studies does translate to the clinical scenario, with an average left-right twist ROM of 2.11 ± 0.52 degrees for unilateral constructs compared to 0.73 ± 0.32 degrees for bilateral constructs. The authors also reported a reduced effect on adjacent segments with unilateral constructs, as indicated by a reduction in adjacent segment motion [5]. Unfortunately, no outcome data was included in this report making it difficult to determine the clinical implications of these differences in motion characteristics.

There are several limitations to this study. First is the fact that only a single cage was used as opposed to a bilateral intervertebral device, as the use of bilateral cages may have changed the results and would require additional testing. The second limitation of this study is in the implementation of an in vitro cadaveric model. Cadaveric models only allow us to assess immediate stability provided by a construct after surgery but before arthrodesis has occurred. This model will never allow us to directly compare the arthrodesis capability between a unilateral and a bilateral spinal construct. For this reason, it is difficult to extrapolate the results from biomechanical testing to direct clinical outcomes. Due to the inherent condition of a cadaveric model, the lack of biologics can also directly affect spinal fusion competence. Although the sample size for this study is statistically in line with other impactful studies, cadaveric variations may have played a role in various motions seen during each testing stage and thus affect the reproducibility of the study. Lastly, posterior spinal instrumentation can have a major impact on observed segment motion due to the size of screws, rod, and interbody devices implanted during surgery. Variables among the instrumentation could be one potential explanation for the diversity of conclusions seen with regard to the comparison of unilateral and bilateral constructs seen in the literature. Additional biomechanical studies are needed to clarify our understanding of this comparison. Extensive clinical research is required before these biomechanical results can be translated into clinical practice, but it is our hope that this study will serve to demonstrate the idea that there are severe biomechanical differences between unilateral and bilateral PLIF constructs that should be considered when deciding upon the appropriate treatment modality.

## 5. Conclusion

Surgeons must consider a multitude of factors when selecting the appropriate treatment strategy for each patient, including biomechanical, perioperative, and postoperative factors in order to reduce the physical and financial burdens of spinal surgery. Unilateral pedicle screw fixation offers a number of benefits with respect to reducing the morbidity of spinal fusion procedures. However, from a biomechanical perspective, PLIF constructs with bilateral pedicle screw fixation provide superior stability compared to unilateral constructs. This trend was observed during both lateral bending and axial torsion loading conditions. Unilateral spinal fixation may be a viable option in some patients during single-level fusion procedures, but caution should be taken when applying the same doctrine to a multilevel scenario. Further research is needed to garner substantiating conclusions regarding the use of unilateral fixation during multilevel spinal fusion procedures.

## Figures and Tables

**Table 1:** Specimen and device information.

Specimen	Facetectomy	Level	Cage
1	Left	L3/L4	12 × 26 mm
		L4/L5	10 × 22 mm
2	Right	L3/L4	10 × 22 mm
		L4/L5	12 × 26 mm
3	Left	L3/L4	12 × 22 mm
		L4/L5	10 × 22 mm
4	Right	L3/L4	10 × 22 mm
		L4/L5	8 × 26 mm
5	Left	L3/L4	8 × 26 mm
		L4/L5	10 × 22 mm
6	Right	L3/L4	12 × 22 mm
		L4/L5	12 × 26 mm

**Table 2:** FE-loading descriptive statistics.

Stage	Flexibility parameters	Raw				Normalized			
		Mean	Std. deviation	95% LCL	95% UCL	Mean	Std. deviation	95% LCL	95% UCL
Intact	ROM	6.87	0.92	4.85	8.89	1.00	—	—	—
	NZ	1.47	0.28	0.86	2.08	1.00	—	—	—
Facetectomy	ROM	7.22	0.88	5.28	9.15	1.14	0.07	0.98	1.29
	NZ	1.41	0.26	0.84	1.99	0.91	0.10	0.68	1.13
Unilateral	ROM	2.89	0.46	1.88	3.90	0.44	0.03	0.37	0.51
	NZ	0.71	0.18	0.30	1.11	0.67	0.16	0.31	1.02
Bilateral	ROM	1.22	0.16	0.87	1.57	0.32	0.12	0.04	0.59
	NZ	0.27	0.07	0.11	0.43	0.52	0.33	-0.21	1.24
Unilateral+cage	ROM	2.51	0.44	1.54	3.48	0.42	0.06	0.28	0.56
	NZ	0.64	0.18	0.24	1.03	0.67	0.25	0.12	1.22
Bilateral+cage	ROM	1.20	0.14	0.89	1.52	0.34	0.13	0.05	0.63
	NZ	0.21	0.06	0.07	0.34	0.31	0.14	0.00	0.63

FE: results of flexion-extension testing. Raw values indicate motion measured in degrees while normalized values represent the amount of motion as a percentage of intact values.

**Table 3:** LB-loading descriptive statistics.

Stage	Flexibility parameters	Raw				Normalized			
		Mean	Std. deviation	95% LCL	95% UCL	Mean	Std. deviation	95% LCL	95% UCL
Intact	ROM	8.13	0.68	6.64	9.63	1.00	—	—	—
	NZ	1.35	0.18	0.96	1.75	1.00	—	—	—
Facetectomy	ROM	8.55	0.71	7.00	10.10	1.05	0.02	1.00	1.11
	NZ	1.62	0.21	1.15	2.09	1.24	0.14	0.94	1.54
Unilateral	ROM	5.23	0.48	4.17	6.28	0.65	0.05	0.54	0.77
	NZ	1.42	0.18	1.03	1.81	1.12	0.15	0.79	1.45
Bilateral	ROM	1.74	0.35	0.98	2.50	0.24	0.06	0.11	0.37
	NZ	0.43	0.15	0.09	0.76	0.41	0.17	0.02	0.79
Unilateral+cage	ROM	4.83	0.56	3.59	6.07	0.59	0.05	0.48	0.70
	NZ	1.34	0.23	0.83	1.84	0.98	0.11	0.74	1.23
Bilateral+cage	ROM	1.78	0.38	0.94	2.62	0.24	0.06	0.1	0.38
	NZ	0.41	0.11	0.16	0.66	0.36	0.12	0.09	0.63

LB: results of lateral bending testing. Raw values indicate motion measured in degrees while normalized values represent the amount of motion as a percentage of intact values.

**Table 4:** AT-loading descriptive statistics.

Stage	Flexibility parameters	Raw				Normalized			
		Mean	Std. deviation	95% LCL	95% UCL	Mean	Std. deviation	95% LCL	95% UCL
Intact	ROM	3.63	0.42	2.70	4.55	1.00		—	—
	NZ	0.86	0.17	0.48	1.23	1.00			
Facetectomy	ROM	4.53	0.51	3.40	5.66	1.26	0.14	0.95	1.58
	NZ	1.05	0.20	0.61	1.49	1.49	0.28	0.86	2.11
Unilateral	ROM	2.67	0.36	1.86	3.47	0.74	0.10	0.52	0.97
	NZ	0.63	0.14	0.32	0.93	0.89	0.20	0.46	1.33
Bilateral	ROM	1.48	0.16	1.12	1.83	0.41	0.04	0.31	0.51
	NZ	0.26	0.05	0.15	0.36	0.36	0.07	0.21	0.52
Unilateral+cage	ROM	2.46	0.27	1.86	3.07	0.69	0.08	0.52	0.85
	NZ	0.66	0.09	0.45	0.86	0.69	0.08	0.52	0.85
Bilateral+cage	ROM	1.64	0.19	1.22	2.07	0.46	0.05	0.34	0.58
	NZ	0.38	0.06	0.25	0.51	0.55	0.08	0.36	0.73

AT: results of axial-torsion testing. Raw values indicate motion measured in degrees while normalized values represent the amount of motion as a percentage of intact values.

## Data Availability

All present data can be seen in Figure 3.
